# One- and Two-Electron Oxidations of β-Amyloid_25-35_ by Carbonate Radical Anion (CO_3_^•−^) and Peroxymonocarbonate (HCO_4_^−^): Role of Sulfur in Radical Reactions and Peptide Aggregation

**DOI:** 10.3390/molecules25040961

**Published:** 2020-02-20

**Authors:** Antonio Francioso, Alessia Baseggio Conrado, Carla Blarzino, Cesira Foppoli, Elita Montanari, Simone Dinarelli, Alessandra Giorgi, Luciana Mosca, Mario Fontana

**Affiliations:** 1 Department of Biochemical Sciences, “Sapienza” University of Rome, 00185 Rome, Italy; a.baseggioconrado@dundee.ac.uk (A.B.C.); carla.blarzino@uniroma1.it (C.B.); Alessandra.giorgi@uniroma1.it (A.G.); 2 Departament of Organic Chemistry, Instituto Universitario de Bio-Orgánica “Antonio González”, University of La Laguna, La Laguna, 38296 Tenerife, Spain; 3 Molecular and Clinical Medicine, University of Dundee, Ninewells Hospital and Medical School, Dundee DD1 9SY, UK; 4 Institute of Molecular Biology and Pathology, CNR National Research Council-Rome, 00185 Rome, Italy; Cesira.foppoli@uniroma1.it; 5 Department of Drug Chemistry and Technology, “Sapienza” University of Rome, 00185 Rome, Italy; Elita.montanari@uniroma1.it; 6 Institute for Structure of Matter, CNR National Research Council-Rome, 00133 Rome, Italy; simone.dinarelli@artov.ism.cnr.it

**Keywords:** peroxymonocarbonate, carbonate radical anion, β-amyloid, methionine sulfoxide, sulfur centered radical, reactive sulfur species, sulfur radical cation

## Abstract

The β-amyloid (Aβ) peptide plays a key role in the pathogenesis of Alzheimer’s disease. The methionine (Met) residue at position 35 in Aβ C-terminal domain is critical for neurotoxicity, aggregation, and free radical formation initiated by the peptide. The role of Met in modulating toxicological properties of Aβ most likely involves an oxidative event at the sulfur atom. We therefore investigated the one- or two-electron oxidation of the Met residue of Aβ_25-35_ fragment and the effect of such oxidation on the behavior of the peptide. Bicarbonate promotes two-electron oxidations mediated by hydrogen peroxide after generation of peroxymonocarbonate (HCO_4_^−^, PMC). The bicarbonate/carbon dioxide pair stimulates one-electron oxidations mediated by carbonate radical anion (CO_3_^•−^). PMC efficiently oxidizes thioether sulfur of the Met residue to sulfoxide. Interestingly, such oxidation hampers the tendency of Aβ to aggregate. Conversely, CO_3_^•−^ causes the one-electron oxidation of methionine residue to sulfur radical cation (MetS^•+^). The formation of this transient reactive intermediate during Aβ oxidation may play an important role in the process underlying amyloid neurotoxicity and free radical generation.

## 1. Introduction

Alzheimer’s disease (AD) is one of the most disabling dementia disorders among the elderly, characterized by progressive loss of memory and cognitive functions. Neurofibrillary tangles of tau protein, neuronal loss, and amyloid plaques are the hallmarks of the disease [1,2]. The major constituent of the amyloid plaques is a 39–43 amino acid peptide named β-amyloid peptide (Aβ) which, through conformational modifications, becomes prone to form aggregates—from soluble oligomers or protofibrils to insoluble large fibrils—and is therefore responsible for various pathological effects [3,4]. Amyloid formation is generally associated with the AD clinical manifestations and there are many reports of Aβ peptides being toxic to neuronal cells [5,6,7]. Although the central role of Aβ in the pathogenesis of the disease is undisputed, the precise mechanism(s) of action and the nature of the toxic species remain to be identified [8].

The damage caused by oxidative stress may play an important role in the initiation and progression of AD. It has been shown that the AD brain is subject to increased oxidative stress [9,10] and that increased Aβ deposits are associated with the sites where neurodegeneration and oxidative stress coexist [11]. According to this, in the Aβ-associated oxidative stress model of neurodegeneration, the peptide is directly responsible for free radical-mediated damage to neuronal membrane systems, leading to subsequent neuronal loss [12,13,14]. Different studies have suggested that the toxic effects of Aβ involve its own oxidation and free radical generation, ascribing a critical role to the methionine (Met) residue at 35 position in the Aβ peptide [14,15,16]. Met-35 is one of the most susceptible residues to be oxidized [17], especially under oxidative stress conditions [18] and is essential for peptide neurotoxicity [19]. In fact, when it is replaced by norleucine or cysteine [19,20] or when it is sequestered within a lipid environment [21], or when it is lacking, as in Aβ_1-28_, the peptide loses its neurotoxic properties.

**Scheme 1:**
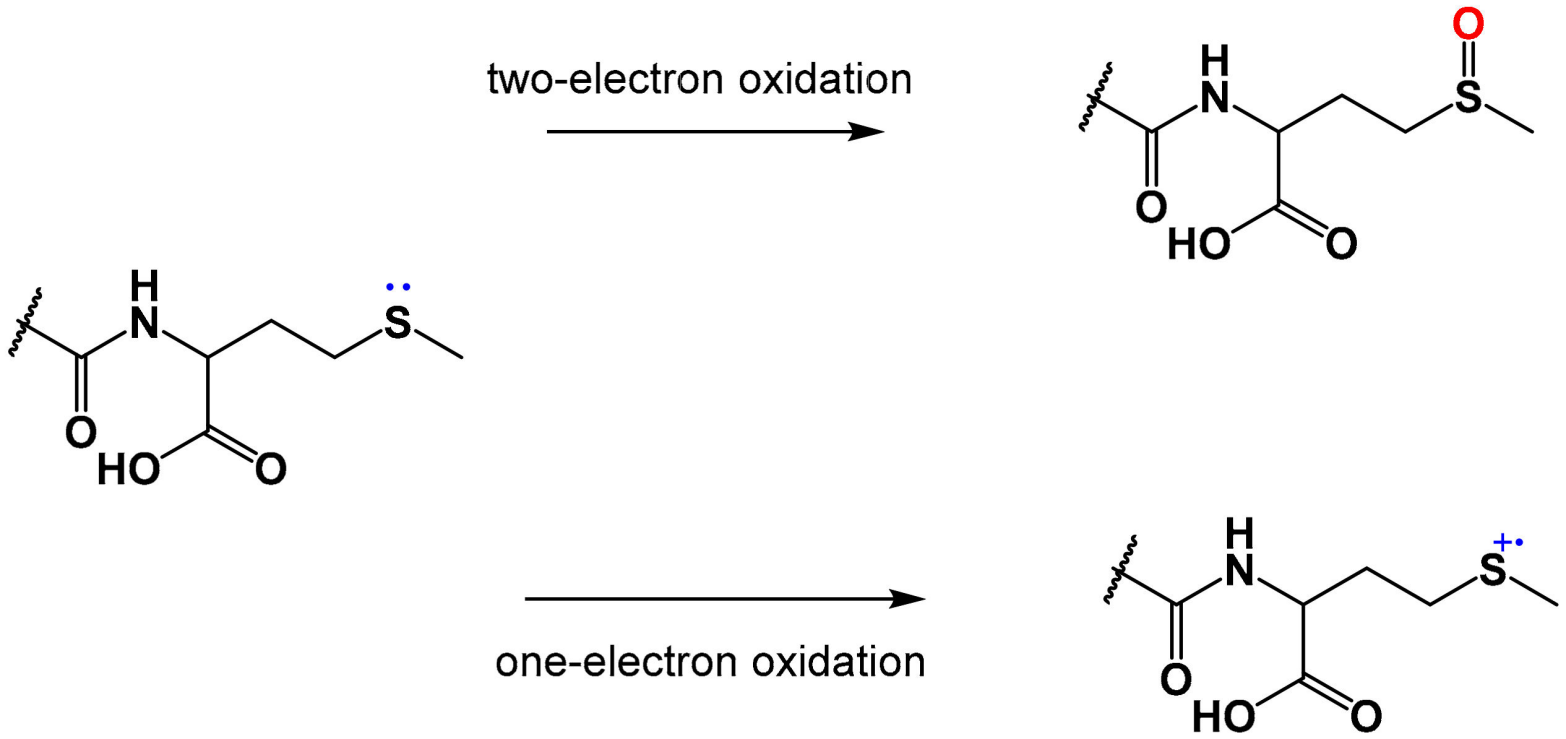
One- and two-electron oxidations of Aβ_25-35_ methionine residue on the thioether sulfur.

In the oxidative event that occurs on Met-35 residue, the thioether sulfur can undergo a two- or one-electron oxidation (Scheme 1). The two-electron oxidation leads to the production of methionine sulfoxide (MetSO) [22], that could be further reduced to Met for the intervention of MetSO reductase, an enzyme whose activity is reduced in AD brains [23], or further oxidized to Met sulfone. Instead, the one-electron oxidation of the thioether sulfur leads to the formation of a positive charged sulfur radical (MetS^•+^) [24].

In order to better elucidate the biochemical process underlying amyloid neurotoxicity and free radical generation, we investigated the oxidative modifications of Aβ by two oxidants derived from the main physiological buffer, the bicarbonate/carbon dioxide pair: peroxymonocarbonate, HCO_4_^−^ (PMC), that catalyzes a two-electron oxidation, and carbonate radical anion (CO_3_^•−^), which is a one-electron oxidant. The experiments were performed by using Aβ_25-35_, a fragment of the full length peptide Aβ_1-42_, containing 11 amino acids and presenting Met-35 as C-terminal residue, which has been shown to be more rapidly toxic and to cause more oxidative damage than the native full-length peptide [25]. Regarding the oxidant species, PMC was generated by the reaction of bicarbonate with hydrogen peroxide [26], while carbonate radical anion was generated by SOD/H_2_O_2_ system in the presence of bicarbonate [27,28]. Products derived from Aβ_25-35_ oxidation were analyzed by HPLC and mass spectrometry and the effects of such oxidation on the aggregation of the peptide were determined by means of spectrofluorimetry, atomic force microscopy, and dynamic light scattering.

## 2. Results

### 2.1. Oxidation of Aβ_25-35_ Fragment by PMC (HCO_4_^−^)

**Figure 1:**
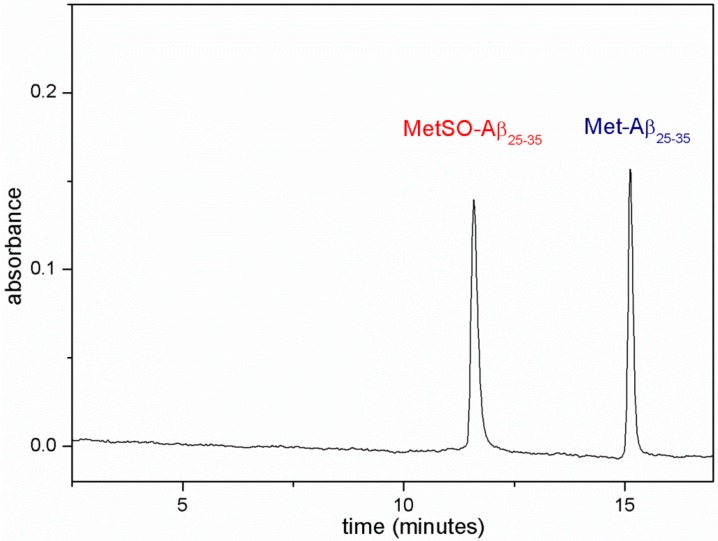
HPLC profile of Aβ_25-35_ incubated for 120 min with peroxymonocarbonate (PMC or *HCO_4_*^−^*)*. 10 μM Aβ_25-35_ was allowed to react with peroxymonocarbonate at 0.8 mM final concentration in 0.1 M phosphate buffer, pH 7.4, containing 0.1 mM DTPA. After 120 min at room temperature, aliquots of incubation mixture were analyzed by HPLC.

The oxidation of Aβ_25-35_ fragment by PMC was evaluated by HPLC. The chromatographic profiles of Aβ_25-35_ solution were incubated for 120 min with HCO_4_^−^ evidenced, in addition to the peak corresponding to the parent Aβ_25-35_ fragment (Met-Aβ_25-35_) at 15 min retention time (RT), the formation of a new peak (MetSO-Aβ_25-35_) with a RT of 11 min (Figure 1).

**Figure 2:**
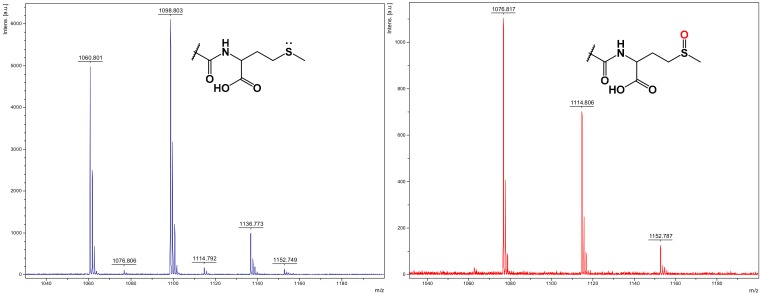
Mass spectra (*m*/*z*) obtained by MALDI-ToF MS analysis related to Aβ_25-35_ (**left**) and the same peptide with the methionine residue oxidized to methionine sulfoxide (**right**). 1060 and 1076 *m*/*z* correspond to Met-Aβ_25-35_ and MetSO-Aβ_25-35_, respectively. The peaks at 1098, 1136, 1114, and 1152 *m*/*z* are related to their +K [M + 39] and +2K [M + 76] *m*/*z* adducts.

Taking into account that the only residue susceptible to be oxidized in the fragment is the Met located at 35 position [18] and the oxidative reactions mediated by PMC proceed through a bi-electronic mechanism [22], the production of the Aβ_25-35_ fragment with the Met residue oxidized to methionine sulfoxide (MetSO-Aβ_25-35_) is expected. In order to confirm this, eluted compounds from HPLC analysis corresponding to the two peaks were collected and analyzed by mass spectrometry. Mass spectra of the two fractions are shown in Figure 2. The two main molecular ions, at m/z = 1060 and 1076, correspond respectively to Aβ_25-35_ and the same peptide with an additional oxygen atom, confirming the formation of MetSO residue.

**Figure 3:**
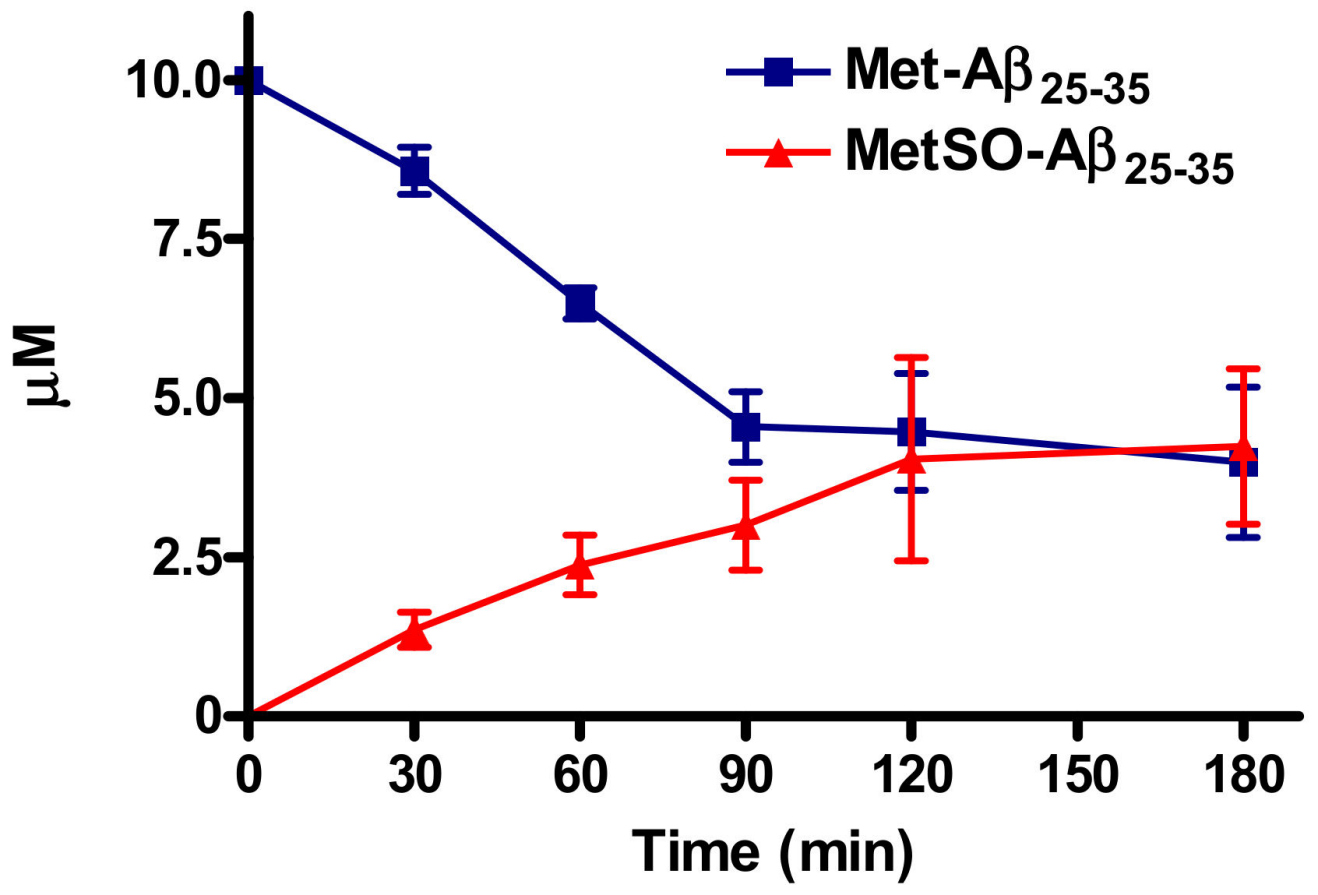
Time course of Aβ_25-35_ oxidation by peroxymonocarbonate. To a reaction mixture containing 10 μM Aβ_25-35_ in 0.1 M phosphate buffer, pH 7.4, and 0.1 mM DTPA, peroxymonocarbonate was added at 0.8 mM final concentration. At various time intervals of incubation at 37 °C, the reaction was stopped by addition of catalase (400 U/mL) and the reaction products were analyzed by HPLC.

The time-dependent conversion of Aβ_25-35_ to MetSO-Aβ_25-35_ by the bi-electronic oxidation was monitored by HPLC analysis. Figure 3 shows the concomitant Met-Aβ_25-35_ diminution and MetSO-Aβ_25-35_ increase.

**Figure 4:**
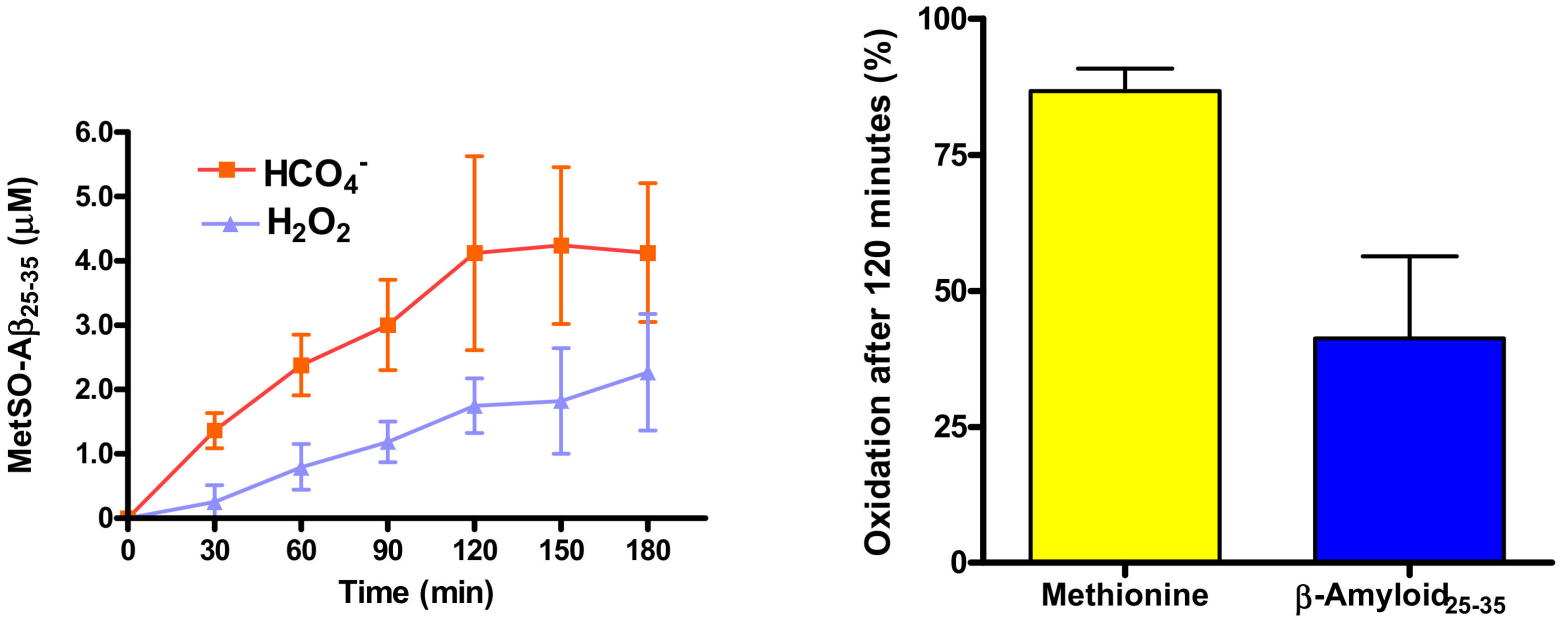
Time course of Aβ_25-35_ oxidation by peroxymonocarbonate or hydrogen peroxide (**left**). Aβ_25-35_ and L-methionine oxidation by peroxymonocarbonate at 120 min (**right**). To reaction mixture containing 10 μM Aβ_25-35_ or 10 μM L-methionine in 0.1 M phosphate buffer, pH 7.4, and 0.1 mM DTPA, peroxymonocarbonate was added at 0.8 mM final concentration. Hydrogen peroxide was 5 mM. At various time intervals of incubation at 37 °C, the reaction was stopped by addition of catalase (400 U/mL) and the reaction products analyzed by HPLC.

MetSO-Aβ_25-35_ formation was also evidenced by HPLC analysis when the peptide was incubated with hydrogen peroxide. The oxidation ability of hydrogen peroxide was lower than that of PMC. In fact, an amount of 41% of amyloid was found to be oxidized after 120 min incubation with PMC, while only 18% was oxidized after the same incubation time with hydrogen peroxide (Figure 4, left).

The sensitiveness to oxidation by HCO_4_^−^ of the Met residue in the Aβ_25-35_ peptide was compared to that of free L-Met in the same experimental conditions. Figure 4 (right) shows that the free amino acid was oxidized to a higher extent compared to the Met residue of the peptide.

### 2.2. Oxidation of Free Methionine and Aβ_25-35_ by CO_3_^•−^ (SOD/H_2_O_2_/Bicarbonate System)

**Figure 5:**
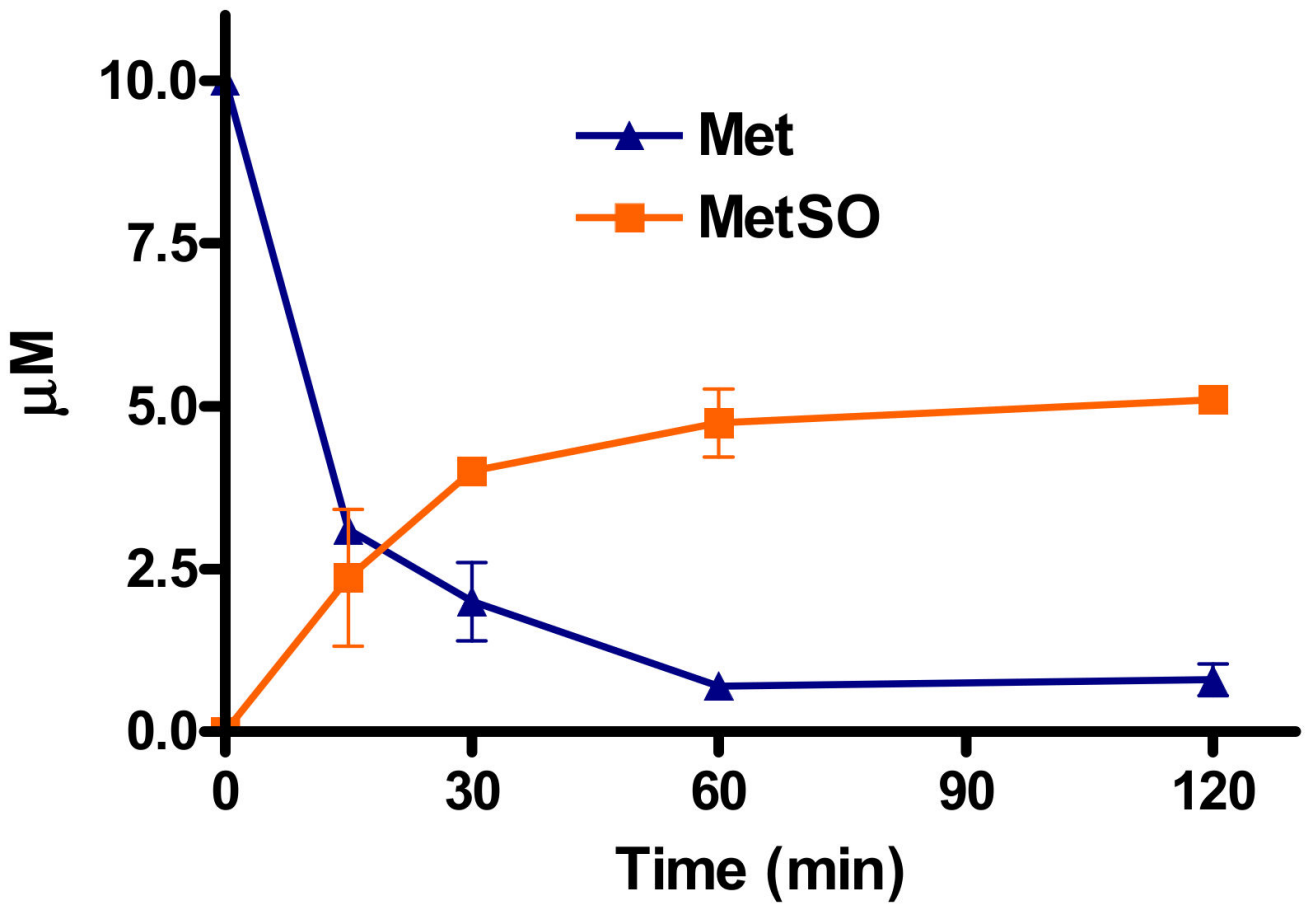
Oxidation of free L-methionine by carbonate radical anion. 10 µM L-methionine was incubated at 37 °C with 31 µM SOD, 25 mM sodium bicarbonate, 0.1 mM DTPA in 0.1 M phosphate buffer, pH 7.4. Reaction was started by the addition of 1 mM H_2_O_2_. At various time intervals, the reaction was stopped by addition of catalase (400 U/mL) and the reaction products analyzed by HPLC.

Oxidative reactions mediated by SOD/H_2_O_2_ system in the presence of bicarbonate proceeded through a mono-electronic mechanism by which the highly reactive carbonate radical anion (CO_3_^•−^) is formed. The effect of HCO_3_^−^/CO_2_ pair on the oxidation of free L-Met in the presence of SOD/H_2_O_2_ was indirectly evaluated quantifying the production of MetSO by HPLC. As shown in Figure 5, free Met was almost completely consumed after 60 min incubation in the presence of SOD/H_2_O_2_/bicarbonate system. However, only a fraction (about 50%) was transformed in MetSO. It can be rationally assumed that the remaining part was oxidized by CO_3_^•−^ through a mono-electronic mechanism to its radical cation form (MetS^•+^), and then degraded to volatile sub-products, such as ammonia, carbon dioxide, and methanthiol, undetectable by HPLC analysis [29,30,31].

On the other hand, in the absence of HCO_3_^−^/CO_2_, the SOD/H_2_O_2_ system determined the full oxidation of L-Met to its sulfoxide. This can be explained by the fact that in these conditions the reaction of SOD with hydrogen peroxide generates ^•^OH radical that inactivates the enzyme [32]; therefore, the observed oxidation was due exclusively to a two-electron mechanism mediated by H_2_O_2_ excess (not shown).

When the Aβ_25-35_ was incubated with the SOD/H_2_O_2_/bicarbonate system under the same experimental conditions, the peak corresponding to the peptide completely disappeared in HPLC chromatogram, but no new peak appeared.

**Scheme 2:**
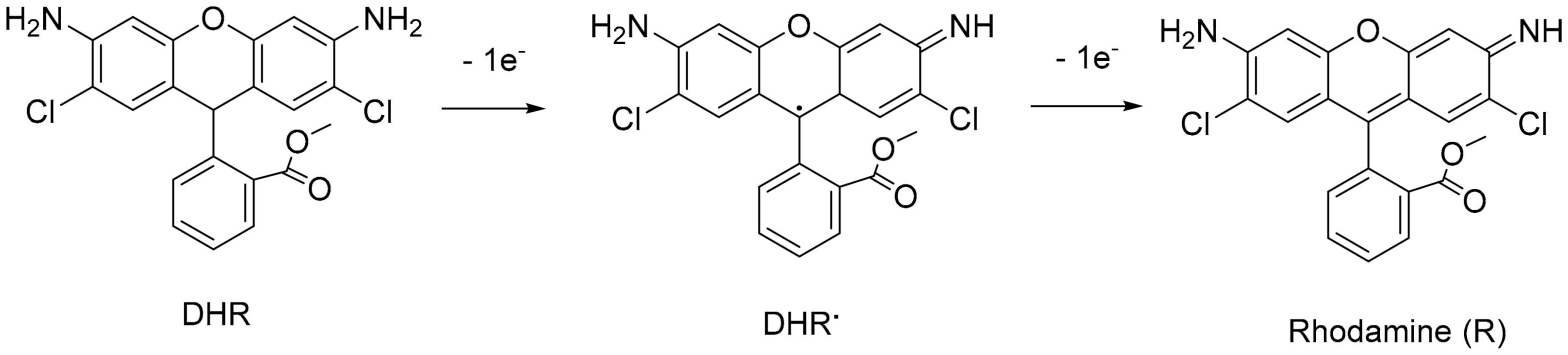
Two-step mono-electronic oxidation of dihydrorhodamine-123 (DHR).

Therefore, the evaluation of one-electron Aβ_25-35_ oxidation was indirectly performed by determining the rate of dihydrorhodamine-123 (DHR) oxidation through mono-electronic mechanism (Scheme 2).

In the presence of CO_3_^•−^, DHR is oxidized first to DHR^•^ radical, and subsequently another electron is abstracted to produce fully oxidized rhodamine (R):

DHR + CO_3_^•−^ → DHR^•^ + CO_3_^2−^

DHR^•^ + CO_3_^•−^ → R + CO_3_^2−^

**Figure 6:**
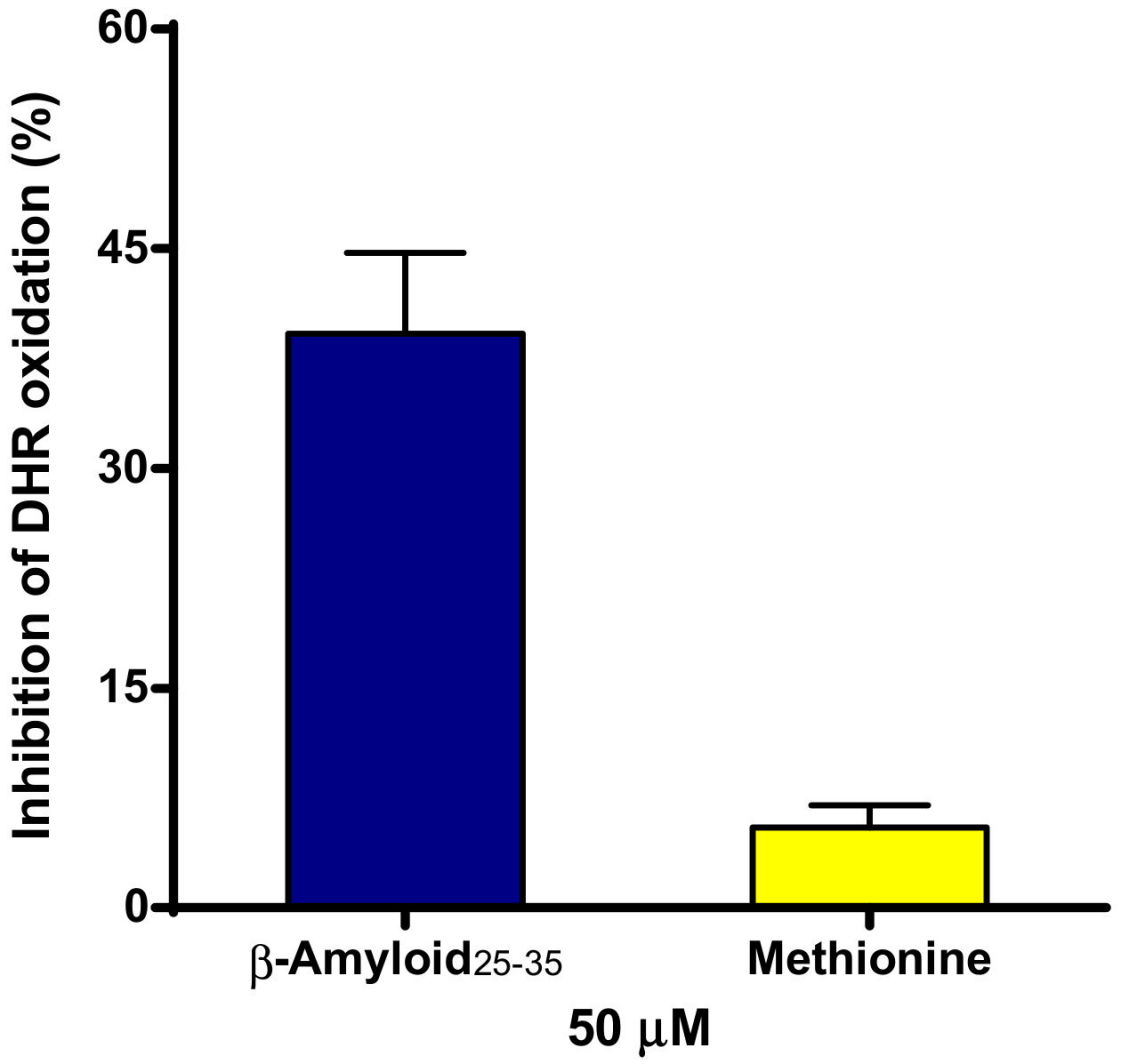
Effect of Aβ_25-35_ and L-Met on DHR oxidation mediated by carbonate radical anion. Aβ_25-35_ or L-methionine (50 µM final concentration) was added to a solution containing 25 µM DHR, 31 µM SOD, 0.1 mM DTPA, and 25 mM sodium bicarbonate in 0.2 M phosphate buffer, pH 7.4. Reaction was started by the addition of 1 mM H_2_O_2_. Oxidized DHR was quantified spectrophotometrically at 500 nm.

Interestingly, Aβ_25-35_ acts as a “scavenger”, competing with DHR in the reaction with carbonate radical anion generated by the SOD/H_2_O_2_/bicarbonate system and showed a much higher ability than free Met in inhibiting the carbonate radical anion-mediated DHR oxidation (Figure 6). The scavenging activity of the free amino acid was very low and a 100-fold Met concentration higher than that of peptide was required to obtain a similar reduction of DHR oxidation rate (not shown).

**Figure 7:**
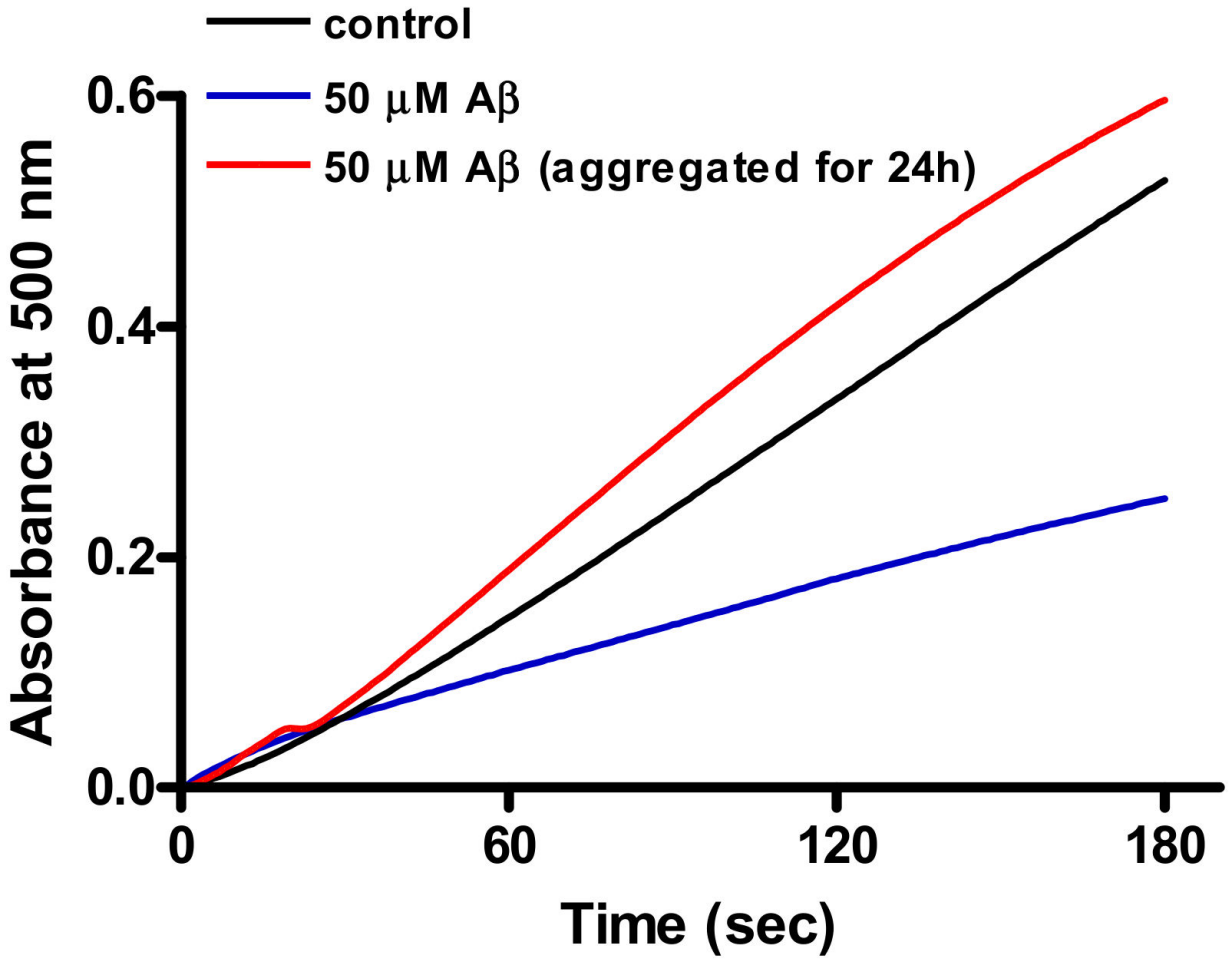
Effect of different aggregation states on the reaction rate of DHR oxidation by CO_3_^•−^. Freshly dissolved Aβ_25-35_ (blue line) and aggregated Aβ_25-35_ (red line) after 24 h incubation. Aβ_25-35_ (50 µM final concentration) was added to a solution containing 25 µM DHR, 31 µM SOD, 0.1 mM DTPA, and 25 mM sodium bicarbonate in 0.2 M phosphate buffer, pH 7.4. Reaction was started by the addition of 1 mM H_2_O_2_. DHR oxidation rate was measured spectrophotometrically at 500 nm.

Noteworthily, the ability of the amyloid to protect DHR from oxidation is exclusive for the non-aggregated peptide. When Aβ_25-35_ was incubated for longer times in DTPA and phosphate buffer (aggregation-favoring conditions), it failed to protect DHR from one-electron oxidation by CO_3_^•−^. Rather, the oxidation rate of DHR in the presence of aggregated peptide was even enhanced compared to the control. Figure 7 shows the kinetics of DHR oxidation in the presence of two different states of Aβ_25-35_ aggregation. After 24 h, during which the peptide was allowed to stay in aggregating conditions, the oxidation rate of DHR was 1.3 fold higher than that of control. The same behavior with lower radical scavenging effects on reaction kinetics was observed using ABTS as a reagent instead of DHR by monitoring the formation of ABTS^+•^ radical cation (not shown).

### 2.3. Effect of One-Electron and Two-Electron Oxidation on Aβ_25-35_ Aggregation

The effect of the two different oxidative modifications of Aβ_25-35_ exerted by HCO_4_^−^ or CO_3_^•^^−^ on Aβ_25-35_ aggregation were investigated by means of spectrofluorimetry (Thioflavin-T assay), dynamic light scattering (DLS), and atomic force microscopy (AFM).

**Figure 8:**
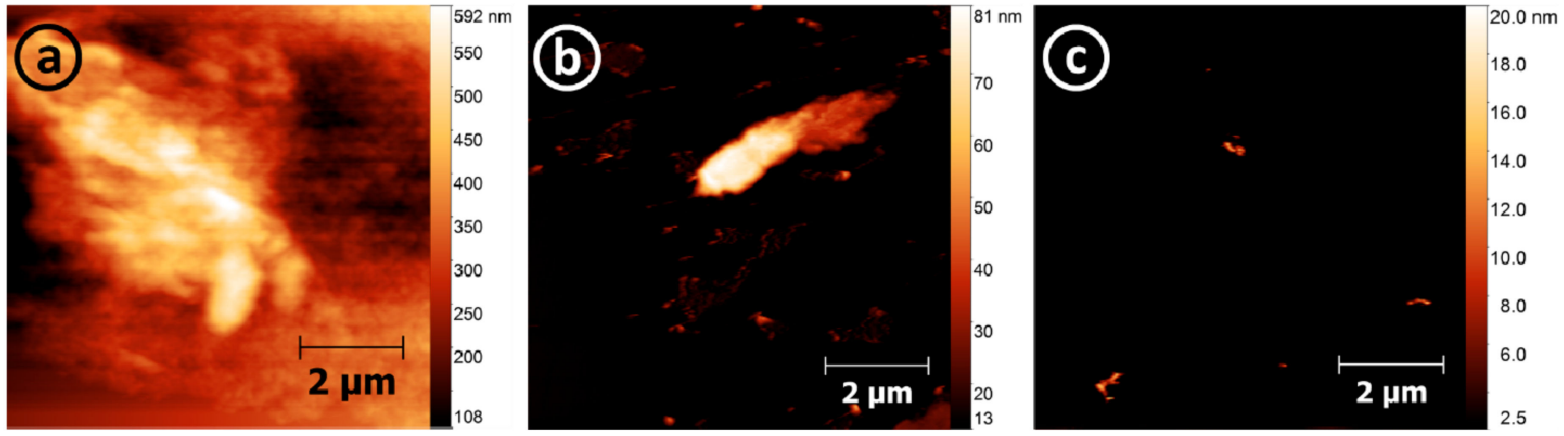
Atomic force microscopy (AFM) typical morphologies of aggregated Aβ_25-35_ in the presence or absence of HCO_4_^−^ (two-electron oxidant) or CO_3_^•−^ (one-electron oxidant) after 24 h aggregation. The aggregated amyloid (**a**) sample present almost everywhere amorphous structures and globular aggregates of several microns in size. The one-electron reaction (**b**) sample is similar to the aggregated one in terms of dimensions of the aggregates but their presence is diminished. Two-electron oxidation sample (**c**), instead, present smaller and scattered structures of elongated nature, in this latter sample very rarely were observed amorphous structures of several hundreds of nanometers in size.

A major consideration needs to be taken into account while using AFM to assess the structures of Aβ samples prepared with the drop casting method on mica: although this deposition technique is quite common and easy to apply, it confers a high heterogeneity to the sample, hampering a quantitative statistical analysis of the observed structures [33]. However, the high resolution of the technique can provide, under some circumstances, a clear idea of the sample properties, i.e., it can be clearly observed whether there are substantial differences among the samples [33]. Figure 8 reports typical images of the specimens, which clearly present morphological differences. As shown in panel (a) in the blank sample there are huge amorphous aggregates of several microns in size and many structures in the order of several hundreds of nanometers that look globular. Panel (b) reports the typical landscape of the one-electron oxidation sample that appears similar to the previous one in terms of co-presence of big aggregates and smaller globular structures. In this case, however, the presence of aggregates of several microns of diameter is reduced and the globular structures ranges from one to some hundreds of nanometers. From an atomic microscopic point of view, this sample looks similar to the previous aggregated Aβ_25-35_ but observed one step before that the huge aggregation process takes place. Finally, in panel (c) of Figure 8, a representative image of the two-electron oxidation sample is shown. In this case the topography is dramatically different, the sample appears scattered, and none of the huge aggregates discussed above have been found, more precisely no structures with diameter of one micron were found. This sample is mainly constituted of small elongated structure of the nanometer range in length, that resemble the fibrillary structure of amyloid fibrils maybe at the very first steps of the aggregation process. It should be noticed that one single fibril per field of view was imaged.

**Figure 9:**
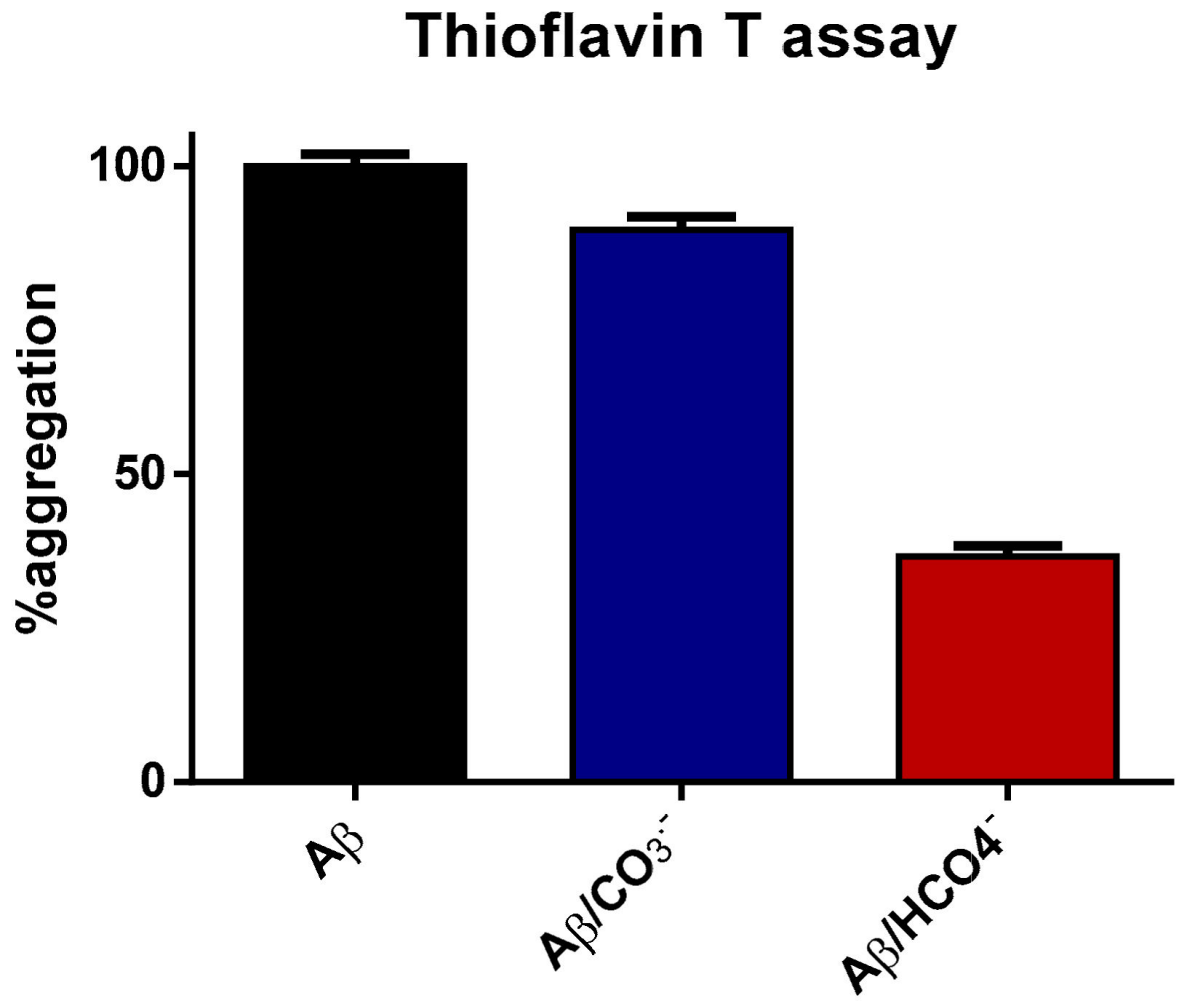
Thioflavin-T fluorimetric aggregation assay of Aβ_25-35_ in the presence or absence of HCO_4_^−^ (two-electron oxidant) or CO_3_^•^^−^ (one-electron oxidant) after 24 h aggregation.

Thioflavin-T (Th-T) assay revealed that after 24 h incubation (Figure 9) the one-electron oxidation mediated by CO_3_^•^^−^ inhibits peptide aggregation for a 10% with respect to the control aggregated Aβ_25-35_. On the other hand, the PMC oxidation of thioether sulfur to sulfoxide (HCO_4_^−^-mediated two-electron oxidation) induces less than 40% aggregation compared to the fully aggregated peptide.

**Figure 10:**
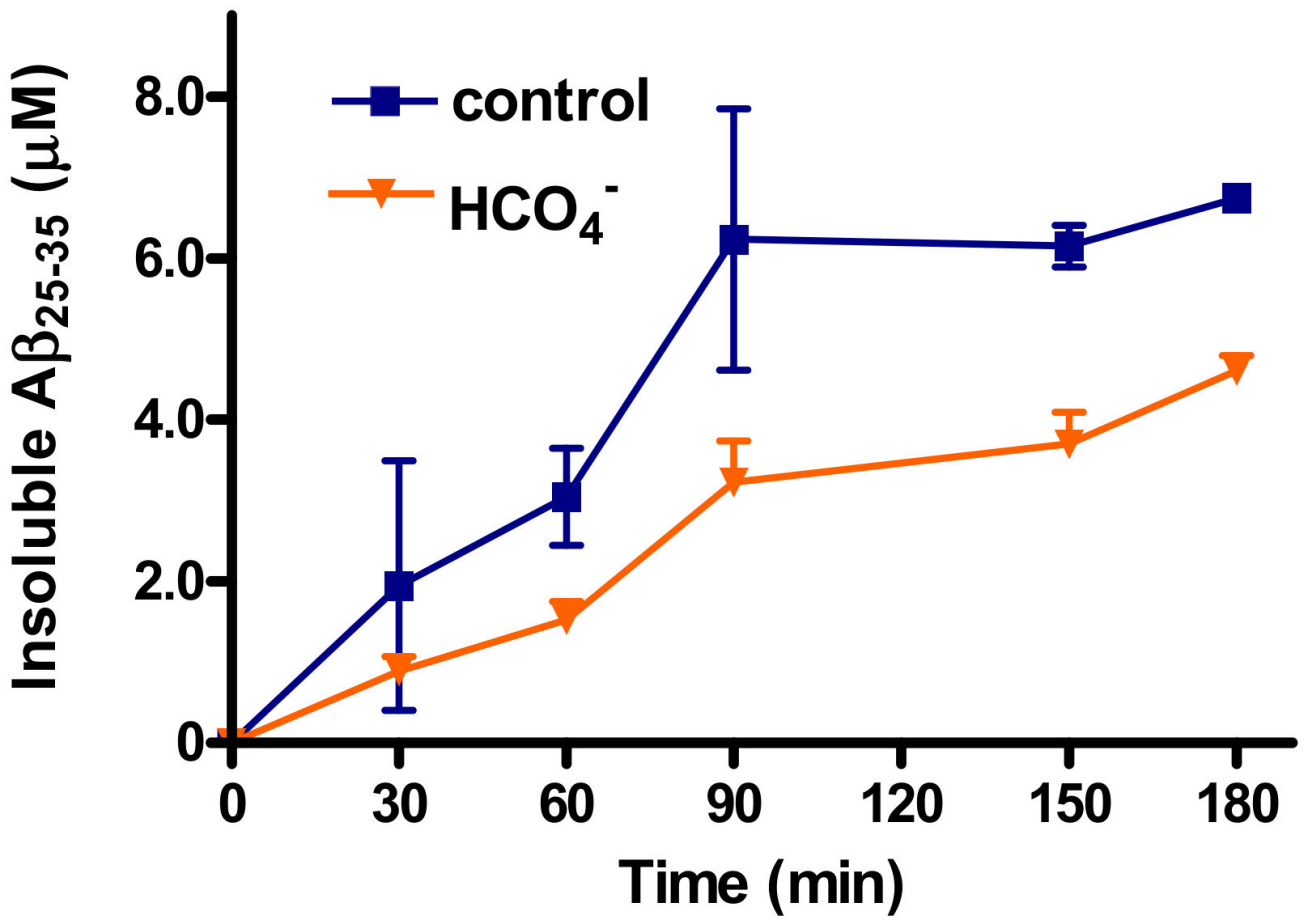
Aβ_25-35_ aggregation in the presence or absence of peroxymonocarbonate. Reaction mixture contained 10 µM Aβ_25-35_ in 0.1 M phosphate buffer, pH 7.4, and 0.1 mM DTPA (squares), when present, peroxymonocarbonate was added at 0.8 mM final concentration (triangles). After various time incubation at 37 °C, the reaction was stopped by addition of catalase (400 U/mL) and the reaction products analyzed by HPLC.

The time-dependent aggregation on HCO_4_^−^ oxidized peptide was also investigated by HPLC. The aggregated amyloid peptide was indirectly quantified by determining the decrease of soluble amyloid peptide. As confirmed by HPLC (Figure 10), the amount of aggregated peptide formed in the presence of HCO_4_^−^ was lower compared to that formed in the absence of the oxidant. A decrease of about 40% was observed after 180 min incubation with PMC.

**Figure 11:**
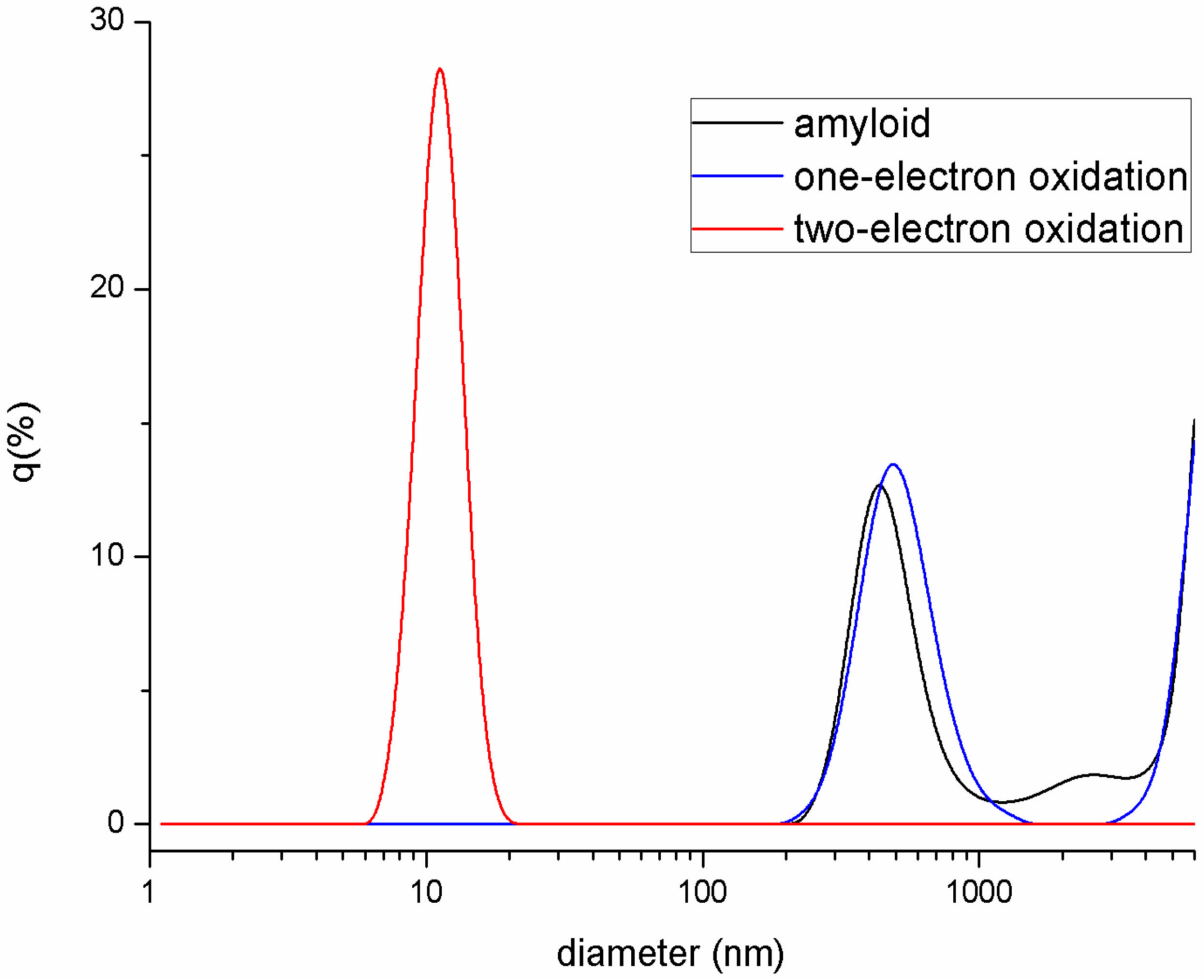
Dynamic light scattering (DLS) analyses of Aβ_25-35_ in the presence or absence of HCO_4_^−^ (two-electron oxidant) or CO_3_^•−^ (one-electron oxidant). Only the sulfoxide formation (red line) mediated by HCO_4_^−^ stops the aggregation process at nano-scale level.

The effect on aggregation and on the tendency of the oxidized peptide to aggregate was explored also by dynamic light scattering experiments. The same samples for AFM and Thioflavin-T analyses were analyzed by DLS after filtering samples through 0.45 µm pore diameters filters. As shown in Figure 11, control Aβ_25-35_ and CO_3_^•^^−^-exposed Aβ_25-35_ (one-electron oxidation) revealed the presence of aggregated particles with micron-scale diameter while the two-electron oxidation of Aβ_25-35_ stops its tendency to form aggregates, maintaining the smaller nano-scale formed particles.

## 3. Discussion

Oxidative damage and amyloid accumulation are the key factors in the pathogenesis of AD. The mechanism by which the amyloid peptide exerts its toxicity is still unknown, but an association between Aβ toxicity and oxidative stress has been suggested and confirmed by several studies [34,35,36].

The Aβ fragment used in this work, the Aβ_25-35_, retains the toxic and aggregation properties of the full-length peptide Aβ_1-42_ [6,25], which is the most abundant form in senile plaques. Indeed, Aβ_25-35_ contains the Met-35 residue that, being the main target of oxidative reactions, is thought to be responsible for the Aβ ability to generate free radicals and oxidative stress.

In this work we show that Aβ_25-35_ undergoes oxidative modifications by the action of oxidants derived from the buffer HCO_3_^−^/CO_2_, i.e., PMC and carbonate radical anion. Indeed, a role of the main physiological buffer in modulating biological oxidations is well established [37,38,39]. Even if both PMC and carbonate radical anion derive from the same system, the reactions mediated by the two oxidants proceed through different mechanisms: HCO_4_^−^ is prevalently a two-electron oxidant, while CO_3_^•−^ is responsible for one-electron oxidations.

PMC can be generated through the equilibrium between bicarbonate and H_2_O_2_ and is capable of oxidizing a variety of organic compounds. Owing to the fact that bicarbonate concentration in tissues under physiological conditions is high (≥25 mM) and, for a fixed hydrogen peroxide concentration, the rate of PMC formation is likely to be similar to that expected for hydroxyl radical formation from redox-active iron(II) [40,41], HCO_4_^−^ can be considered an reliable biological oxidant.

Our results indicate that PMC is able to oxidize thioether sulfur of Met-35 residue of Aβ_25-35_ to sulfoxide (MetSO). The Met oxidation to MetSO is well known and its presence in proteins is an established marker of oxidative stress [18,42,43]. Mechanisms of oxidation of sulfides and free methionine by PMC have been widely studied by Richardson and co-workers [44,45]. We observed that PMC is more potent than hydrogen peroxide as concerns the Aβ oxidation, in accordance with some experimental evidence on the ability of the bicarbonate/carbon dioxide system to promote oxidation, peroxidation and nitration of several biological targets in the presence of hydrogen peroxide [37,46]. In these conditions, PMC is considered the chemical species responsible for the increment of oxidative reactions. Although the formation of HCO_4_^−^ from hydrogen peroxide and bicarbonate in aqueous solution at neutral pH is a relatively low process (k = 10^−2^ M^−1^sec^−1^), this value is influenced by the presence of proteins and lipids [40], indicating that PMC can be physiologically generated from hydrogen peroxide at high speed in biological tissues rich in proteins, lipids, and bicarbonate/CO_2_.

Interestingly, our experiments show that Aβ_25-35_ two-electron oxidation counteracts the tendency of peptide to aggregate. This is in accordance with previous reports, indicating that the oxidation of Met-35 also alters the physical properties of the peptide, such as aggregation tendency, leading to a different oligomerization profile with significant attenuation of trimers and tetramers formation [47] and reduction of fibril assembly [48]. Additionally, recent molecular dynamics simulation experiments of Brown et al. [49] are consistent with a decrease in aggregation rate when Met-35 of Aβ_1-40_ is oxidized to sulfoxide. Moreover, it has been observed that Met oxidation of Aβ generates very different fiber morphologies compared to un-oxidized Aβ, with much shorter (<500 nm) fragmented fibers [50]. Noteworthily, also the inhibition of fibrillation of α-synuclein is mediated by methionine oxidation. It has been suggested that α-synuclein completely oxidized to its Met residues shows a reduced propensity to form amyloid fibrils, probably related to the interference of MetSO residues in oxidized protein to form ordered β-type secondary structures [51].

The fact that oxidized amyloid possesses attenuated aggregating capacity validates the hypothesis that oxidative stress in AD brain likely induces toxicity in a manner independent from fibril formation. This theory is also supported, considering the outcomes of the one-electron oxidation of Aβ_25-35_ by carbonate anion radical (CO_3_^•−^). This strong oxidant acts by both electron transfer and hydrogen abstraction mechanisms to produce radicals from the oxidized targets [39] and is able to oxidize a broad series of compounds of biochemical interest [52], including sulfur amino acids of proteins [53] in which it adds to the sulfur atom, according to reactions [54]:

MetS + CO_3_^−^ → MetS^•^CO_3_^−^

MetS^•^CO_3_^−^ → MetS^+^ + CO_3_^2−^

In our study CO_3_^•−^ has been generated by SOD/H_2_O_2_/HCO_3_^−^ system. Bicarbonate anion, owing its small size, can easily reach SOD active site, where is oxidized to CO_3_^•−^ by hydroxyl radical (^•^OH) generated by the enzyme in the presence of hydrogen peroxide:

SOD-Cu(II) + H_2_O_2_ → SOD-Cu(I) + O_2_^−^ + 2H^+^

SOD-Cu(I) + H_2_O_2_ → SOD-Cu(II)^•^OH + OH^−^

SOD-Cu(II)^•^OH + HCO_3_^−^ → SOD-Cu(II) + H_2_O + CO_3_^−^

To investigate the susceptibility of Aβ_25-35_ fragment to mono-electronic oxidation by carbonate anion radical, we carried experiments using dihydrorhodamine-123 as a target of oxidative reaction by CO_3_^•−^. We observed that the presence of Aβ_25-35_ determined a net decrease of DHR oxidation rate, due to the fact that the peptide competes with DHR for the CO_3_^•−^. Instead, if Aβ_25-35_ is preventively incubated for long times in favoring aggregation conditions, the DHR oxidation rate results also 1.3 fold higher than that in absence of peptide. Therefore, the reactivity of amyloid with carbonate radical anion confers to the peptide an antioxidant effect until it is in unaggregated form. On the contrary, under conditions that favor the peptide aggregation, the antioxidant capacity is lost and the peptide becomes pro-oxidant. This behavior suggests the formation of radical intermediates able to promote the oxidative processes.

Compared to free methionine, Aβ_25-35_ was much more efficient as competitor of DHR for the CO_3_^•−^-mediated oxidation (Figure 7). The low scavenger activity of free methionine is in accordance with previous reports [52] on the greater susceptibility of amino acid residues into proteins respect to free amino acids to be oxidized by carbonate anion radical. Additionally, our HPLC determinations showed that, when free methionine was allowed to react in the presence of SOD/H_2_O_2_/HCO_3_^−^, it was only partially oxidized through one-electron transfer, and that a fraction was transformed in sulfoxide through two-electron oxidation; on the contrary, CO_3_^•−^ completely oxidized Aβ_25-35_, as demonstrated by the fact that in the same conditions no peak attributable to two-electron oxidation of Met residue appeared.

The pro-oxidant effect of Aβ has been often correlated to its ability to chelate metals, copper above all, due to the presence of three histidine residues in the peptide chain [55,56] and it has been suggested that this ability could explain the presence of elevated concentration of copper and other transition metals in the senile plaques of AD subjects [57]. However, in our experiments, the pro-oxidant effect has been observed despite the presence of DTPA, a strong metal-chelating agent, and even though the truncated peptide we used, Aβ_25-35_, lacks of metal-binding residues of native peptide Aβ_1-42_. It can be therefore hypothesized that the observed pro-oxidant activity could be linked to the high reactivity of Met-35. Indeed, reactivity of methionine residues toward carbonate radical anion is in the order of 10^7^ M^−1^sec^−1^, at least 100 fold greater than that of not-sulfur amino acids [52].

In the presence of CO_3_^•−^, methionine residue undergoes one-electron oxidation, generating a sulfur radical cation. This “sulfur centered” radical is potentially pro-oxidant, since is able to remove hydrogen atoms from protein and lipids, causing protein oxidation and lipid peroxidation [58]. At pH 7.4, the C-terminal of Aβ_25-35_, corresponding to methionine residue, is in the form of carboxylate anion. The negatively charged oxygen could favor the oxidation of thioether sulfur and the stabilization of the derived cation radical. Moreover, the MetS^•+^ radical cation in the full-length peptide Aβ_1-42_ can be stabilized through bond formation with either the oxygen or the nitrogen atoms of adjacent peptide bond [59]. Likely, this radical remains stabilized also during the aggregation process, therefore making the peptide able to trigger oxidative reactions.

## 4. Materials and Methods

### 4.1. Reagents and Chemicals

Aβ_25-35_ fragment (NH_2_- and COOH-terminal free peptide), superoxide dismutase (SOD) from bovine erythrocytes (EC 1.15.1.1), catalase, diethylenetriaminepentaacetic acid (DTPA), and Thioflavin-T were obtained from Sigma (St. Louis, MO, USA). l-methionine and hydrogen peroxide (30%) were purchased from Fluka (Buchs, Switzerland). All other chemicals were of the highest purity commercially available. H_2_O_2_ concentration was verified using UV absorption at 240 nm (ε = 43.6 M^−1^cm^−1^) [60]. All solutions and buffers were prepared with distilled water purified in a Millipore (Burlington, MA, USA) Milli-Q system and contained DTPA to avoid metal-dependent oxidative reactions.

### 4.2. Synthesis of PMC (HCO_4_^−^)

0.9 mL of 0.5 M sodium bicarbonate were added to 0.1 mL of 0.5 M hydrogen peroxide and the solution was left to equilibrate at room temperature for 10 min. In these conditions, the concentration of formed HCO_4_^−^ calculated by the equation: [HCO_4_^−^] = 0.31 × [H_2_O_2_] [HCO_3_^−^], where 0.31 is the value of k of equilibrium for the HCO_4_^−^ formation [39], results to be 7.75 mM.

### 4.3. Two-Electron Oxidation of Aβ_25-35_ Fragment and L-Methionine by PMC

To reaction mixture containing 10 µM Aβ_25-35_ or 10 µM L-methionine in 0.1 M phosphate buffer, pH 7.4, and 0.1 mM DTPA, PMC was added at 0.8 mM final concentration. After incubation at 37 °C for various times, the reaction was stopped by addition of catalase (400 U/mL) and the reaction products were analyzed by HPLC and mass spectrometry (Waters, Milford, MA, USA; Bruker Daltonics, Bremen, Germany).

### 4.4. One-Electron Oxidation of Aβ_25-35_ Fragment and L-Methionine by SOD/H_2_O_2_ System

To reaction mixture containing 10 µM Aβ_25-35_ or 10 µM L-methionine in 0.1 M phosphate buffer, pH 7.4, and 0.1 mM DTPA, SOD was added at 31 µM final concentration, in the presence or absence of 25 mM NaHCO_3_. The reaction was started by the addition of hydrogen peroxide at 1 mM final concentration. After incubation at 37 °C for various times, the reaction was stopped by addition of catalase (400 U/mL) and the reaction products were analyzed by HPLC.

### 4.5. HPLC Analysis

HPLC analysis of methionine and methionine sulfoxide (Met-SO), the two-electron oxidation product, was performed with a Waters–Millipore Chromatograph (Milford, MA, USA) equipped with a model 600 pump, a model 600 gradient controller, and a Waters 474 scanning fluorescence detector (Milford, MA, USA). Samples were derivatized with *o*-phthalaldehyde-2-mercaptoethanol (OPA) prior to injection [61] and applied on a Symmetry C18 column (5μ, 4.6 mm × 250 mm). Elution was performed using as mobile phases: A) 50 mM sodium acetate buffer, pH 5.5-methanol (80:20, *v*/*v*) B) 50 mM sodium acetate buffer, pH 5.5-methanol (20:80, *v*/*v*), flow rate 1mL/min, at room temperature. The elution gradient was linear, from 100% A to 100% B in 30 min. The fluorescence of eluates was monitored at 340 nm (λ_ex_) and 450 nm (λ_em_). The retention time of L-methionine was 21 min and concentrations were calculated from a standard curve.

For analysis of Aβ_25-35_ and its two-electron oxidation product, the UV Waters photodiode array detector was used. The chromatography was carried out on a Nova-Pak C18 column (4 μ, 3.9 mm × 150 mm). Elution was performed using as mobile phases: (A) 50 mM sodium phosphate buffer, pH 3.0-acetonitrile (90:10, *v*/*v*) (B) acetonitrile-water (50:50, *v*/*v)*, flow rate 1mL/min, at room temperature and with a linear gradient from 100% A to 100% B in 30 min. The absorbance of eluates was monitored at 214 nm. The retention time of Aβ_25-35_ was 15 min and its concentration was calculated from a standard curve using the Millenium 32 software (Waters). The same curve was used to determine oxidized Aβ_25-35_ concentration, assuming the same absorbance at 214 nm for either Aβ_25-35_ and its oxidized form.

### 4.6. Purification of the Product of Two-Electron Oxidation of Aβ_25-35_ Fragment by PMC

Then, 10. µM Aβ_25-35_ was allowed to react with PMC at 0.8 mM final concentration in 0.1 M phosphate buffer, pH 7.4, containing 0.1 mM DTPA. After 120 min at room temperature, aliquots of incubation mixture were loaded on Nova-Pak C18 column and subjected to HPLC in the conditions previously described. The eluates, monitored at 214 nm, corresponding to the product of two-electron oxidation of Aβ_25-35_, with a retention time of 11 min, were collected and analyzed by MALDI-ToF MS spectrometry (Bruker Daltonics, Bremen, Germany) [62].

### 4.7. Mass Spectrometry

The peptide mixtures were loaded directly onto an appropriate MALDI target plate with 1 μL of α-cyano-4-hydroxy-trans-cinnamic acid matrix solution (10 mg/mL) in 70% acetonitrile containing 0.1% TFA (*v*/*v*). MALDI-ToF MS analyses were performed by an AutoFlex II (Bruker Daltonics, Bremen, Germany) equipped with a 337 nm nitrogen laser and operating in reflector mode. Mass data were obtained by accumulating several spectra from laser shots with an accelerating voltage of 20 kV.

### 4.8. Effect of L-Methionine and Aβ_25-35_ Fragment on Dihydrorhodamine-123 (DHR)

Stock solution of 5 mM DHR in acetonitrile was prepared and stored in the dark at −20 °C. Appropriate aliquots of L-methionine or Aβ_25-35_ (50 µM final concentration) were added to a solution containing 25 µM DHR, 31 µM SOD, 0.1 mM DTPA, and 25 mM sodium bicarbonate in 0.2 M phosphate buffer, pH 7.4. Reaction was started by the addition of 1 mM H_2_O_2_. Oxidized DHR was quantified spectrophotometrically at 500 nm (ε = 78,000 M^−1^cm^−1^) [63] with a UV-Vis Perkin-Elmer (Waltham, MA, USA).

### 4.9. Thioflavin-T (ThT), Atomic Force Microscopy (AFM), and Dynamic Light Scattering Analyses (DLS)

One-electron oxidation, two-electron oxidation, and control Aβ_25-35_ (in PBS buffer) samples were incubated for 24 h at room temperature. An aliquot of each sample was used for ThT, AFM, and DLS experiments. Thioflavin T (ThT) binding assay was performed as reported [64]. Then, 50 μL of each sample was added to a fresh 5 μM solution of Thioflavin-T (dissolved in 50 mM glycine solution, pH 8.5) to rich a final 10 μM Aβ_25-35_ concentration. Fuorescence was measured on a SPEX-Fluoromax spectrofluorometer (Horiba Scientific, Horiba Ltd., Kyoto, Japan) with λ_ex_ = 440 nm and λ_em_ = 485 nm. The results are presented as mean and S.D. of three separate measurements.

The samples for the AFM characterization were prepared by the casting method, by manually dropping 5 μL of sample solution onto freshly cleaved mica and then air dried overnight, as previously described [33]. The AFM images were acquired using a home-designed microscope, described in detail elsewhere [65], operating in contact mode, in air, at room temperature and constant 30% relative humidity. The AFM tips chosen were made of silicon nitride (Veeco, New York, NY, USA) with tip of asymmetric pyramidal shape and nominal radius on 10 nm. During the imaging the vertical force was maintained below 1 nN in order to not damage the samples. The acquired images (8 × 8 micron, 600 × 600 points) were then treated using the freeware software Gwyddion (www.gwyddion.net).

For DLS experiments each samples was filtered onto 0.45 μm filters to retain insoluble and over micron scale aggregates and analyzed after one hour from filtration. The DLS measurements were taken using a HORIBA LB-550 instrument at a 650 nm Laser diode wavelength (5 mW), with a temperature control capacity set at 25 °C. To ensure accurate readings, the final distribution was taken from an accumulation of 100 individual DLS collections of three individual aliquots for each sample.

### 4.10. Statistics

Results are expressed as means ± SEM for at least three separate experiments performed in duplicate. Graphics and data analysis were performed using GraphPad Prism 4 software.

## 5. Conclusions

Although the origin of oxidative damage in AD is not yet fully understood, our experiments confirm that amyloid peptide is liable to be oxidized at level of methionine residue and that the effects of such oxidation vary depending whether one- or two-electron mechanisms are implied. While bi-electronic oxidation of the thioether sulfur of Met-35 to sulfoxide determines an attenuation of aggregation capacity of the peptide, as demonstrated by our and other authors’ experiments [19], the one-electron oxidation leads to the formation of sulfur radical cation (MetS^•+^)^.^ The latter, like other radicals with sulfur in high oxidation states that can be originated in vivo in oxidative stress conditions [66], has oxidizing properties similar to ROS and is able to trigger and spread oxidative reactions. Therefore, though the sulfur-containing biomolecules have antioxidant activity, the transient formation of these reactive species during redox reactions may have a patho-physiological relevance [67]. This is a further confirmation of the model of free radical-based neurotoxicity of Aβ in AD, supported by numerous lines of evidence [16,24], according which the peptide initiates free radical processes resulting in protein oxidation, lipid peroxidation, reactive oxygen species formation, and cellular dysfunctions leading to neuronal death [16].

Finally, it is noteworthy that the oxidative agents used in this work, i.e., PMC and carbonate radical anion, derive in vivo from the main physiological buffer, the bicarbonate/CO_2_ pair, which can promote either mono-electronic oxidations mediated by peroxynitrite, SOD, xanthine oxidase, or bi-electronic oxidations mediated by hydrogen peroxide. The high bicarbonate concentration physiologically present in tissues makes the importance of the two species very considerable. The described processes may occur in physiological conditions, and the inclusion of these species within the more prominent biological oxidants can give new prompts in the comprehension and control of the mechanisms of many pathologies.

It should be emphasized that free radicals and oxidants are currently considered as important mediators in the response of various signaling molecules and pathways involved in physiologic and pathologic processes. A better understanding of cellular oxidative mechanisms, many of which are likely to be modulated by the ubiquitous bicarbonate/carbon dioxide pair, can be very helpful for the elucidation of these interrelated processes.

## Abbreviations

Aβ	β-amyloid
AD	Alzheimer’s disease
DTPA	diethylenetriaminepentaacetic acid
DHR	dihydrorodhamine-123
MetSO	methionine sulfoxide
MetS^•+^	methionine sulfur radical cation
PMC	peroxymonocarbonate (HCO_4_^−^)
